# A predictive model for first-trimester pregnancy inception after IVF-ET based on multimodal ultrasound evaluation of endometrial receptivity

**DOI:** 10.1186/s12880-022-00863-w

**Published:** 2022-09-04

**Authors:** Jianmei Liao, Shuping Yang, Keyue Chen, Huijun Chen, Fan Jiang, Weina Zhang, Xuebin Wu

**Affiliations:** 1 Department of Ultrasound, Zhangzhou Affiliated Hospital of Fujian Medical University, Zhangzhou, 363000 Fujian China; 2 Reproductive Medicine Center, Zhangzhou Affiliated Hospital of Fujian Medical University, No. 59 Shengli Road, Zhangzhou, 363000 Fujian China

**Keywords:** Three-dimensional ultrasound, Endometrial receptivity, Predictive model, First-trimester pregnancy

## Abstract

**Background:**

In-vitro fertilization-embryo transfer (IVF-ET) is a commonly used assisted reproductive technology. Its success depends on many factors, including endometrial receptivity. Endometrial receptivity can be evaluated by ultrasound, endometrial biopsy, and magnetic resonance imaging. Compared with the latter two methods, ultrasound has the advantages of wide availability, non-invasiveness, and low cost. Three-dimensional (3D) ultrasound imaging examines endometrial thickness, morphology, and blood vessels, which are associated with the success of embryo implantation. However, there are no reports of endometrial receptivity assessment by 3D ultrasound. Therefore, we aimed to evaluate endometrial receptivity using 3D ultrasound and construct a predictive model for first-trimester pregnancy inception following IVF-ET.

**Methods:**

We performed a prospective observational study on infertile women who underwent IVF-ET between December 2019 and February 2021. These women had 3D ultrasound evaluations, measuring endometrial thickness, volume, pattern, morphology, peristalsis, uterine artery blood flow index, sub-endometrial blood flow index, and distribution pattern. We recorded the occurrence of first-trimester pregnancies in these women. Using Akaike information criterion (AIC) and backward stepwise regression, a first-trimester pregnancy prediction model was constructed based on the minimum AIC value and validated internally and externally.

**Results:**

111 women were enrolled, with 103 included in the analysis. Univariate and multiple logistic regression analyses showed that endometrial thickness and vascularization flow index (VFI) were independent factors associated with the occurrence of a pregnancy. The final prediction model corresponding to the minimum AIC value (65.166) was Y = − 6.131–0.182endometrial thickness + 0.542endometrial volume + 4.374VFI + 0.132age. In the test set, modeling cohort, and external validation cohort, the model showed satisfactory differentiation, with C index of 0.841 (95%CI 0.699–0.817), 0.727 (95%CI 0.619–0.815), and 0.745 (95%CI 0.671–0.840), respectively. The *Hosmer–Lemeshow* goodness of fit tests reported *P* = 0.865, 0.139, and 0.070, respectively, indicating a high agreement with the actual IVF-ET outcome. This model reached the highest diagnostic efficiency (sensitivity 88.9%, specificity 75%, Youden index 0.639) at a diagnostic cut-off value of ≥ 0.360.

**Conclusions:**

The predictive model based on endometrial receptivity evaluations by 3D ultrasound had high diagnostic efficiency and could be a simple and effective tool to predict first-trimester pregnancy inception after IVF-ET.

## Background

The incidence of infertility has gradually increased in recent years, with reports that approximately 48 million couples live with infertility globally [1]. In-vitro fertilization-embryo transfer (IVF-ET) is the current mainstream assisted reproductive technology (ART). The success of an IVF-ET depends on certain factors, including endometrial receptivity. Endometrial receptivity refers to the window of implantation (WOI) during which the uterus allows embryo implantation to occur [2] and is considered one of the most important factors [3]. A satisfactory endometrial receptivity refers to the state in which the endometrium allows the embryo to adhere, with subsequent corresponding endometrial changes for the implantation [4]. Appropriate clinical interventions can optimize endometrial receptivity to achieve an ideal conception state and a successful pregnancy outcome. Therefore, evaluating endometrial receptivity has become critical in selecting these clinical interventions.

Endometrial receptivity can be evaluated using different methods, including endometrial biopsy, magnetic resonance, and ultrasound. An endometrial biopsy can examine the endometrium at the histological level [5], but it is an invasive method with a high incidence of complications and is not widely accepted in clinical practice. Magnetic resonance imaging has certain advantages in clarifying the thickness of the endometrium and its relationship with the myometrium [6], but the investigation is expensive and time-consuming. Ultrasound is widely used in clinical practice because of its convenience, non-invasiveness, and low cost. Three-dimensional (3D) ultrasound imaging can evaluate endometrial thickness, pattern, and vascularity and comprehensively assess the female pelvis before ART [7]. It has become one of the promising new ultrasound technologies. Some studies have suggested that endometrial thickness could be used as a surrogate indicator of endometrial receptivity, but its correlation with live birth rate requires further investigation [8]. In addition, other studies have reported that endometrial peristalsis was related to the success of embryo implantation [9]. Virtual Organ Computer-aided Analysis (VOCAL) may improve the reliability of endometrial measurements by manually mapping a multi-plane standard view of the endometrium and subendometrial vascularity [10].

However, there is no report on the application of 3D ultrasound to evaluate endometrial receptivity and pregnancy inception comprehensively. Therefore, we used 3D ultrasound and VOCAL to examine the endometrial pattern and blood flow in this study. We then explored their relationships with pregnancy inception to identify independent risk factors for adverse IVF-ET outcomes and construct a first-trimester pregnancy predictive model.

## Methods

### Study design and participants

We performed a prospective observational study and enrolled infertile women who received IVF treatments at the Reproductive Medicine Center of Zhangzhou Affiliated Hospital of Fujian Medical University, China, from December 2019 to February 2021. The hospital’s ethics committee approved the study protocol. All the study participants signed the informed consent document.

Inclusion criteria were reproductive age women with (1) clinically diagnosed infertility, failure to achieve a pregnancy after 12 months or more of regular unprotected sexual intercourse; (2) normal ultrasound examination of the uterine morphology, with no endometrial polyps, submucous myomas, or uterine synechiae. (3) one or two high-quality embryos available for fresh embryo transfer during the IVF treatment cycle; and (4) had 3D ultrasound examination of the uterus on the day of embryo transfer (ET).

Exclusion criteria included those women with (1) prior uterine surgery; (2) chronic estrogen or progesterone treatment; (3) male factor infertility; or (4) secondary infertility.

### Instruments and methods

Instrument: GE Voluson E8 RIC5-9-D intracavity probe, frequency 4-9 MHz.

Methods: Two-dimensional ultrasound, color Doppler flow imaging (CDFI), power Doppler imaging (PDI), and 3D ultrasound evaluated endometrial thickness, pattern, morphology, endometrial peristalsis, subendometrial and uterine artery blood flow, and endometrial volume on the day of the ET.

**Fig. 1:**
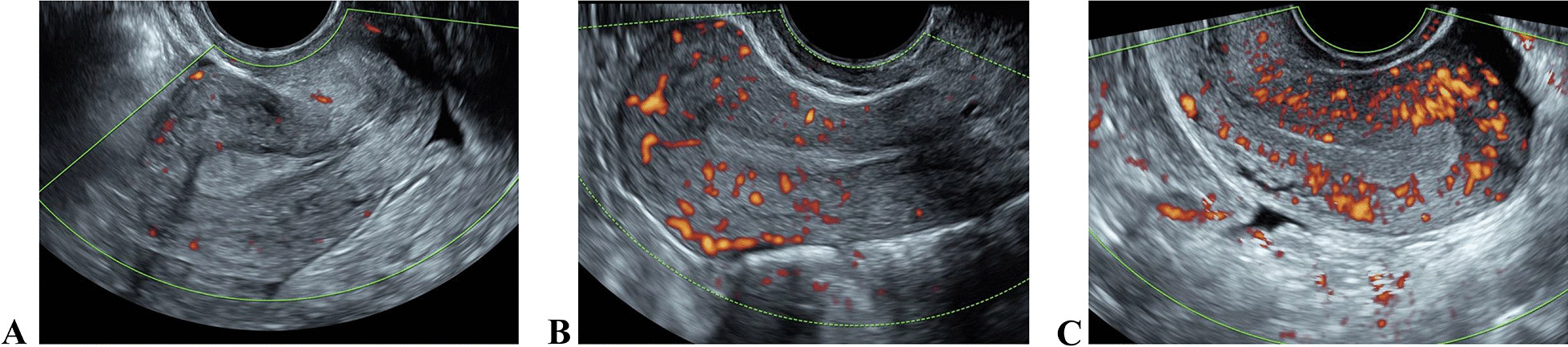
Sub-endometrial blood flow classification. **A** Type I; **B** Type II; **C** Type III

**Fig. 2:**
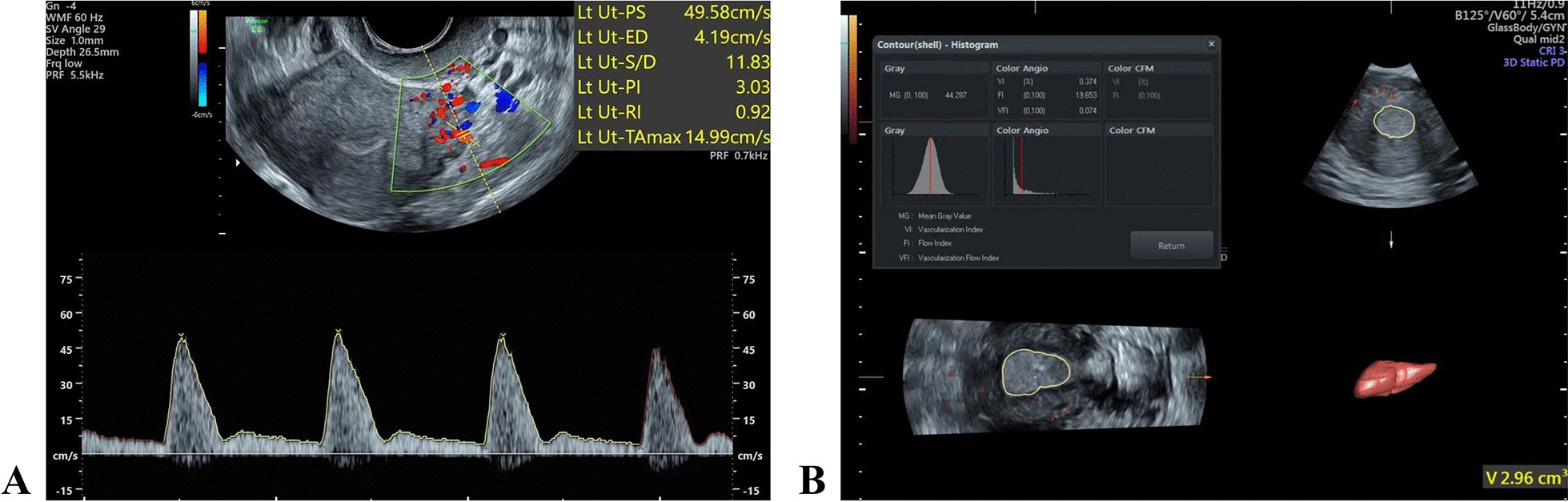
**A** The uterine artery blood flow parameters. **B** Endometrial 3D vascularization parameters

Ultrasound was performed by one senior ultrasonographer on the day of the ET. Endometrial thickness was measured at the thickest area perpendicular to the endometrial midline in the central sagittal plane. Gonen morphological classification of the endometrium was applied as Type A: typical three-layer or multilayered endometrium, with hyperechogenic outer lines and midline and hypoechogenic or anechoic areas between these lines; Type B: homogeneous isoechogenic endometrium, with unclear hyperechogenic endometrial midline; Type C: homogeneous hyperechogenic endometrial midline. Ijland classification of endometrial peristalsis was Type I: no activity; Type II: waves from the cervix to the fundus, Type III: waves from the fundus to the cervix, Type IV: opposing waves starting simultaneously at the cervix and fundus, Type V: random waves starting at various foci [11]. Applebaum classification of sub-endometrial blood flow was, Type I: blood vessels pass through the lateral hypoechogenic band of the endometrium but do not enter the hyperechogenic endometrial rim; Type II: blood vessels cross the hyperechogenic endometrial rim; Type III: blood vessels enter the endometrium [12] (Fig. 1). Pulsed wave Doppler was used for measuring the uterine artery blood flow with an angle of less than 60°. The uterine artery blood flow sampling point was about 2 cm outside the internal cervix. Three to five continuous, stable and consistent flow velocity waveforms were recorded. The systolic peak flow velocity, resistance index, and pulsatility index of the left and right uterine arteries were measured thrice, and the average value was calculated (Fig. 2A).

The ultrasound scan was performed in the following steps: starting the power Doppler, switching to the 3D mode, adopting the multi-plane mode, adjusting the sampling box to completely cover the endometrium, setting the volume angle at 60°, starting the scanning, obtaining the 3D volume data, applying the VOCAL software, and measuring the endometrial volume (volume, V) and the endometrial vascularization parameters, including vascularization index (VI), flow index (FI), and vascularization flow index (VFI) (Fig. 2B).

### IVF-ET procedure and pregnancy determination

Ovum retrieval was scheduled 36 h after intramuscular hCG injection on the hCG day. Embryos were transferred 72 h later. After ovum retrieval, patients were given progesterone orally until 14 days after the ET.

Serum β-hCG and progesterone were measured 14 days after the ET. A color Doppler ultrasound scan was performed 30 days after the ET if increased levels were detected. The presence of an intrauterine gestational sac was considered as the first-trimester pregnancy.

### Statistical analysis

*R version 4.0.5* (http://www.r-project.org/) was used for statistical analysis. Continuous variables were tested for normality by the *Kolmogorov–Smirnov test*. Those with normal distribution are presented as mean ± standard deviation, while those without normal distribution are presented as median [interquartile range]. Categorical classified variables are represented as numbers (percentage).

In the training set of the modeling cohort, the *rms* package was used to perform univariate analysis. The Variables that reached the significant level (*P* < 0.25, Wald test) were included in the multivariate logistic regression analysis to obtain the odds ratio (*OR*) and 95% confidence interval (*CI*) of each independent risk factor. The prediction model corresponding to the minimum AIC value was selected according to the AIC. The *Hosmer–Lemeshow* goodness of fit test was used to test the calibration of the above models. The receiver operating characteristic (ROC) curve calculated the optimal diagnostic cut-off value. The modeling cohort was divided randomly into a training set and a testing set according to the proportion of 7:3. The predictive model was built using the data from the training set. The model was then validated using the testing set and the external validation cohort. A *P* < 0.05 was considered statistically significant.

## Results

### Patient enrollment

A total of 111 patients aged 22–42 years underwent fresh ET during the study period. Three patients were lost to follow-up, and two patients discontinued treatment. Furthermore, three patients had uterine lesions diagnosed by ultrasound examination. Finally, 103 patients were included in the analysis, with an average age of 32.57 ± 4.49 years. The characteristics of the two cohorts are shown in Table 1.

**Table 1:** Characteristics of the modeling cohort and the external validation cohort

	Modeling cohort		External validation cohort	
	Non-pregnancy (n = 40)	Pregnancy (n = 33)	Non-pregnancy (n = 12)	Pregnancy (n = 18)
Age^a^	32.77 (4.95)	32.73 (4.44)	34.25 (4.79)	30.72 (2.63)
Endometrial thickness^a^	11.01 (1.95)	12.08 (2.71)	12.44 (2.78)	12.03 (3.15)
Endometrial volume^a^	4.31 (1.40)	5.21 (1.81)	5.09 (2.07)	5.79 (2.72)
Endometrial morphology^c^				
Type A	5(12.5%)	2 (6.1%)	0 (0.0%)	3 (16.7%)
Type B	26 (65.0%)	28 (84.8%)	8 (66.7%)	13 (72.2%)
Type C	9 (22.5%)	3 (9.1%)	4 (33.3%)	2 (11.1%)
Uterine artery mPI^a^	2.18 (0.38)	2.12 (0.38)	1.97 (0.36)	2.05 (0.50)
Uterine artery mRI^a^	0.81 (0.05)	0.80 (0.05)	0.78 (0.05)	0.79 (0.05)
Uterine artery mS/D^a^	5.62 (1.44)	5.39 (1.38)	5.04 (1.39)	5.01 (1.01)
VI^b^	0.67 [0.21, 1.39]	1.05 [0.63, 2.95]	0.18 [0.16, 0.35]	1.34 [0.64, 3.53]
FI^a^	15.14 (3.00)	16.03 (3.91)	15.07 (3.86)	17.63 (3.23)
VFI^a^	0.09 [0.03, 0.21]	0.17 [0.07, 0.52]	0.03 [0.02, 0.04]	0.30 [0.11, 0.61]
Sub-endometrial blood flow^c^				
Type I	18 (45.0%)	10 (30.3%)	8 (66.7%)	3 (16.7%)
Type II	19 (47.5%)	20 (60.6%)	4 (33.3%)	12 (66.7%)
Type III	3 (7.5%)	3 (9.1%)	0 (0.0%)	3 (16.7%)
Endometrial peristalsis^c^				
Type I	6 (15.0%)	7 (21.2%)	1 (8.3%)	1 (5.6%)
Type II	1 (2.5%)	1 (3.0%)	1 (8.3%)	1 (5.6%)
Type III	2 (5.0%)	3 (9.1%)	0 (0.0%)	1 (5.6%)
Type IV	0 (0.0%)	1 (3.0%)	0 (0.0%)	1 (5.6%)
Type V	31 (77.5%)	21 (63.6%)	10 (83.3%)	14 (77.8%)

n, number of patients; a, mean (standard deviation); b, median [interquartile range]; c, numbers (percentage); *mPI* mean pulsatility index, *mRI* mean resistance index, *mS/D* mean systolic peak flow velocity/ end diastolic velocity

### Univariate analysis and multivariate Logistic regression analysis

Using the *rms* package for univariate analysis, the significant factors (*P* < 0.25, Wald test) were included in the multivariate logistic regression analysis to obtain OR and 95% CI of independent risk factors. The results showed that endometrial thickness and VFI were independent predictors of first-trimester pregnancy inception after IVF-ET(Table 2).

**Table 2:** Univariate analysis and multivariate Logistic regression analysis

	Univariate analysis OR (95% CI)	*P*	Multivariate analysis OR (95% CI)	*P*
Age	0.99 (0.88, 1.11)	0.799	–	–
Endometrial thickness	1.18 (0.92, 1.51)	0.191	0.70 (0.52, 0.94)	0.019
Endometrial volume	1.53 (1.03, 2.28)	0.037	1.54 (0.98, 2.41)	0.062
Endometrial morphology				
Type A	Control	–	Control	–
Type B	6.47 (0.69, 60.68)	0.102	2.58 (0.13,51.8)	0.536
Type C	2.00 (0.13, 29.81)	0.615	7.86 (0.21, 299.19)	0.267
Uterine artery mPI	1.12 (0.24, 5.26)	0.890	–	–
Uterine artery mRI	1.62 (0.00, 2.98e+5)	0.938	–	–
Uterine artery mS/D	1.07 (0.7, 1.63)	0.752	–	–
VI	1.60 (1.03, 2.48)	0.036	–	–
FI	1.10 (0.93, 1.31)	0.27	–	–
VFI	17.08 (1.36, 215.12)	0.028	33.3 (1.6, 692.38)	0.024
Sub-endometrial blood flow				
Type I	Control		Control	
Type II	3.07 (0.91, 10.37)	0.071	0.70 (0.11, 4.44)	0.704
Type III	2.17 (0.24, 19.28)	0.488	0.50(0.02, 11.44)	0.663
Endometrial peristalsis				
Type I	0.83 (0.04, 16.99)	0.906	–	–
Type II	Control			
Type III	2.00 (0.05, 78.25)	0.711	–	–
Type IV	5.76e+6 (0.00, ∞)	0.991	–	–
Type V	0.84 (0.05, 14.57)	0.906	–	–

e+5, 10^5^; e+6, 10^6^; ∞, positive infinity

### Model construction and verification

A full-variable model was constructed, and models were screened according to the AIC criteria. The outcome prediction model corresponding to the minimum AIC value (65.166) was as follows:

$${\text{Y}}\, = \, - {6}.{131}{-}0.{\text{182X}}_{{1}} \, + \,0.{\text{542X}}_{{2}} \, + \,{4}.{\text{374X}}_{{3}} \, + \,0.{\text{132X}}_{{4}} .$$

**Fig. 3:**
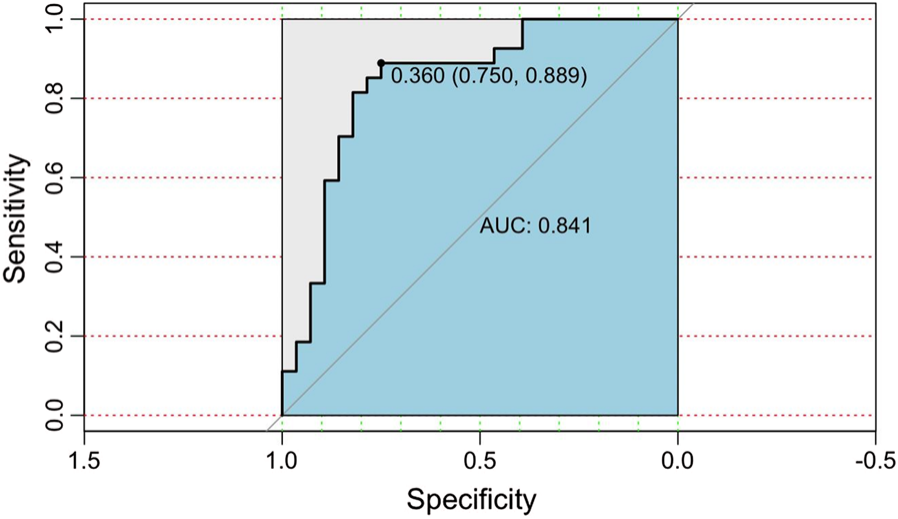
ROC curve

**Fig. 4:**
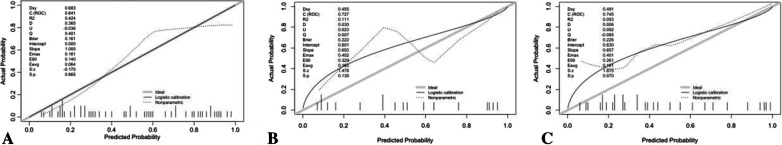
Calibration curve. **A** the training set of the modeling cohort; **B** the testing set of the modeling cohort; C: the external validation cohort

X_1_ is the endometrial thickness (mm). X_2_ is endometrial volume (ml). X_3_ is VFI. X_4_ is age (years). When the diagnostic cut-off value was ≥ 0.360, satisfactory pregnancy inception was considered, with the sensitivity, specificity, and Youden index being 88.9%, 75%, and 0.639, respectively (Fig. 3). The C-index of the model was 0.841 (95%CI 0.699–0.817). The *Hosmer–Lemeshow* goodness of fit test had a *P* = 0.865 (Fig. 4A).

In the testing set, the model C-index was 0.727 (95%CI 0.619–0.815), with the *Hosmer–Lemeshow* goodness of fit test *P* = 0.139. In the external validation cohort, the model C-index was 0.745 (95%-CI 0.671–0.840), with the *Hosmer–Lemeshow* goodness of fit test *P* = 0.070 (Fig. 4B, C).

## Discussion

The quality of the embryo and endometrial receptivity play a synergistic role in reaching a successful pregnancy. In recent years, the evaluation of endometrial receptivity has been studied extensively since endometrial receptivity is a crucial factor in determining the inception of pregnancy. Three-dimensional ultrasound has critical clinical applications. Compared with other examination methods, 3D ultrasound can easily and noninvasively measure various parameters of endometrial receptivity and provide the reference for embryo implantation, growth, and pregnancy outcome. However, few studies have comprehensively evaluated endometrial thickness, endometrial volume, morphology, endometrial artery blood perfusion, and the construction of a pregnancy prediction model by 3D ultrasound. Therefore, we incorporated variables to construct a predictive model to facilitate the clinical evaluation of successful embryo implantation. The predictive model can provide a non-invasive and simple examination method to assess the outcome of IVF-ET.

Maged et al. [13] used two-dimensional transvaginal ultrasound and a 3D power Doppler ultrasound to measure endometrial volume and sub-endometrial blood perfusion in 82 patients undergoing embryo transfer on the hCG day and ET day, respectively. The results showed a statistically significant difference in the endometrial volume between pregnant and nonpregnant groups (*P* < 0.05). However, Shui et al. [14] reported different study results, showing no significant difference in the endometrial volume between the nonpregnant and pregnant groups (*P* > 0.05). Our results showed that endometrial volume differed between the two groups in the univariate analysis but not in the multivariate analysis, suggesting that the endometrial volume was not an independent predictor of pregnancy. Our study showed that endometrial morphology was not a factor associated with pregnancy occurrence (*P* > 0.05), consistent with the results of Martins et al. [3]. Uterine artery parameters (mPI, mRI, and mSD) were not significantly correlated with pregnancy occurrence (P > 0.05), which might be because the uterine artery supplies several vital structures, such as fallopian tubes and ovaries, in addition to the uterus. In this study, endometrial peristalsis was not associated with pregnancy occurrence (*P* > 0.05). Endometrial peristalsis refers to the spontaneous rhythmic peristalsis of the endometrium. The frequency, intensity, and direction of uterine contraction waves change throughout the menstrual cycle [15]. Our study results were inconsistent with Kim et al.’s [16], which suggested that endometrial contraction, cervical orientation, and hyperechogenic endometrium affected the inception of pregnancy; probably due to the failure of ultrasound to measure the uterus's dynamic data at rest and quantify the extent of uterine contractions.

Kasius et al. [17] proposed a positive correlation between pregnancy rates and endometrial thickness. A thicker endometrium could indicate a higher chance of pregnancy. Our results also suggest that endometrial thickness is an independent predictor of pregnancy occurrence (*P* < 0.05). Sardana et al. [18] found an association between pregnancy rates and sub-endometrial blood flow in hormonal replacement FET cycles. Univariate and multivariate logistic regression analyses in the present study found that VFI on the day of transplantation is an independent factor affecting post-transplantation pregnancy (*P* < 0.05), which is consistent with previous studies [17, 18]. Our results confirm the positive correlations between the endometrial thickness and VFI index with the occurrence of a pregnancy.

There is no uniform color Doppler standard or method to evaluate endometrial receptivity in infertile patients. Previous studies only included uterine artery flow index or subintimal blood flow to evaluate the endometrial receptivity [19, 20]. For example, Wang et al*.* assigned patients into three groups for evaluation based on their sub-endometrial blood flow [20]. In order to comprehensively and accurately evaluate endometrial receptivity during an IVF-ET cycle, our study included various endometrial ultrasound measurements. We then screened these measurements and constructed a pregnancy prediction model based on the AIC principle, which avoided underfitting and overfitting of the model [21].

Interestingly, based on the minimum AIC value (65.166), the final model included not only endometrial thickness and VFI but also endometrial volume and age. The model performs well, with a C-index of 0.841 in the training set, indicating that the model had a good distinction. The *Hosmer–Lemeshow* goodness of fit test indicated a high consistency (*P* = 0.865). The model achieved maximum diagnostic efficiency by taking 0.360 as the diagnostic cut-off value. The C-indexes of the model in the testing set and the external validation cohort were 0.727 and 0.745, respectively, indicating a satisfactory prediction of pregnancy following IVF-ET by this model.

In this study, we first evaluated endometrial thickness, endometrial volume, morphology, and endometrial artery blood perfusion by 3D ultrasound and then constructed a predictive model for the inception of a first-trimester pregnancy after IVF-ET. Our results showed that the predictive model had high diagnostic efficiency and could accurately predict pregnancy inception following IVF-ET. However, this study has some limitations, including small sample size and single-center research. VOCAL software requires manual description, which could cause individual variations when measuring the endometrial boundaries. Our model was based on ultrasound examinations. Its accuracy could be affected by the techniques of the different ultrasonographers. Our study results should also be validated under different IVF-ET protocols to test their generalizability.

## Conclusions

The predictive model had excellent consistency and generalizability, a simple and effective method for clinicians to predict pregnancy inception after IVF-ET. Age and endometrial receptivity, as represented by endometrial thickness, volume, and vascularization flow index measured by 3D ultrasound, could successfully predict pregnancy inception following IVF-ET.

## Data Availability

The datasets generated and analyzed during the current study are not publicly available because none of the data types require uploading to a public repository. However, they are available from the corresponding author on reasonable request.

## Abbreviations

3D: Three-dimensional
IVF-ET: In-vitro fertilization-embryo transfer
AIC: Akaike information criterion
WHO: World Health Organization
ART: Assisted reproductive technology
ET: Embryo transfer
VI: Vascularization index
FI: Flow index
VFI: Vascularization flow index
OR: Odds ratio
CI: Confidence interval
ROC: Receiver operating characteristic
